# Two stage posterior surgery using temporary Magnetically Controlled Growing Rod for severe and rigid Adolescent Idiopathic Scoliosis: A retrospective single-centre cohort study

**DOI:** 10.1016/j.bas.2025.105888

**Published:** 2025-11-28

**Authors:** Mauro Spina, Enrico Salvatore D'Agostino, Roberto Giuliani, Francesco Greco, Massimo Balsano

**Affiliations:** a Regional Spinal Department, University and Hospital Trust, Verona, Italy; b Orthopaedics and Traumatology, University and Hospital Trust, Verona, Italy; c Orthopaedic Department, Santa Chiara Hospital, APSS Trento, Italy

**Keywords:** Severe rigid idiopathic adolescent scoliosis, Ponte osteotomies, Magnetically controlled growing rods, Sistema quadro, Three phases correction

## Abstract

**Introduction:**

To evaluate clinical and radiographic outcomes in patients with severe, rigid Adolescent Idiopathic Scoliosis (AIS) (Cobb angle >100°, flexibility index <30 %) treated using a two-stage posterior-only approach with temporary Magnetically Controlled Growing Rods (MCGR).

**Material and methods:**

Between 2019 and 2024, nine patients (eight Lenke 1, one Lenke 3; mean age 15 years; BMI 18.8) underwent staged posterior correction. The first stage included high-density pedicle screw fixation (1.92 screws/vertebra), multiple Ponte osteotomies (mean 4.7), and placement of a concave-side MCGR fixed proximally and distally with a custom construct (“Sistema Quadro”). Postoperative distraction was performed daily for approximately 14 days using an External Remote Controller, achieving a mean rod lengthening of 18.2 mm. The second stage consisted of MCGR removal and definitive posterior spinal fusion.

**Results:**

The main Cobb angle improved from 107.6° to 35.4° (p < 0.0001), corresponding to a mean correction of 65.9 %, obtained in three phases: intraoperative distraction (52.5 %), postoperative lengthening (24.5 %), and final fusion (23 %). Trunk height increased by 9.5 cm and thoracic height by 5.4 cm. Coronal balance improved (25.7 mm–14.4 mm; p = 0.32), as did the clavicle angle (4.4°–0.8°; p = 0.0005). SRS-22 scores rose from 3.3 to 4.4 (p = 0.0011). An inverse correlation was observed between BMI and rod lengthening (PCC = −0.7304; p = 0.026). No complications occurred.

**Discussion and conclusions:**

A two-stage posterior technique utilizing temporary MCGRs, combined with the “*Sistema Quadro*” construct and a *three-phase correction* strategy, offers a safe, effective, and well-tolerated surgical approach for severe, rigid AIS. This method facilitates gradual, controlled deformity correction, optimizes clinical and radiographic outcomes, and minimizes perioperative complications.

## Highlights

• Two-stage posterior-only correction with MCGR used in severe AIS >100°.
• Innovative anchoring construct “Sistema Quadro” improved stability.
• Mean Cobb angle corrected from 107.6° to 35.4° (65.9 % correction).
• Significant thoracic (+5.4 cm) and trunk height (+9.5 cm) gains achieved.
• No neurological or mechanical complications observed in 9 patients.
• SRS-22 scores improved from 3.3 to 4.4 at final follow-up.

## 1 Introduction

The definition of severe and rigid Adolescent Idiopathic Scoliosis (AIS) remains controversial, with no universally accepted diagnostic criteria established in the current literature. AIS is a three-dimensional spinal deformity, and various studies have proposed threshold values for defining severity, with a Cobb angle ranging from >70° to >100°, and a flexibility index ranging from <20 % to <40 % (Traversari et al., 2022; Faldini et al., 2023).

The surgical management of this complex deformity is technically demanding and associated with significant risks. Historically, the most commonly employed strategy was a two-stage approach, consisting of anterior thoracotomy and release followed by posterior instrumentation (Shufflebarger et al., 1991), often with an intermediate period of halo-gravity traction (Savini et al., 1989). Alternatively, a single-stage posterior-only approach involving aggressive three-column osteotomies (3COs), such as Vertebral Column Resection (VCR), has been used (Suk et al., 2002).

In 2006, as an alternative to halo traction, Buchowski et al. (2007) introduced the concept of temporary internal distraction using mechanical growing rods. By applying this technique, Cheng et al. (2012) in 2012 conducted a posterior-only two-stage procedure with temporary internal distraction in a series of 18 severe and rigid spinal deformities (Cobb angle >100°), achieving an average correction rate of 60.4 %. Similarly, Hui Min Hu et al. (2016) reported on 11 cases of extremely severe and rigid scoliosis (mean Cobb angle 148.8°, flexibility <15 %), achieving an average correction of 63 % with a mean interval of 3.5 months between the two stages.

In 2014, Cheung JP et al. (Cheung et al., 2014) proposed a novel method for gradual correction of severe pediatric spinal deformities using magnetically controlled growing rods (MCGR). Subsequently, in 2020, Di Silvestre et al. (Di Silvestre et al., 2020) applied this MCGR technique in a multicenter study of 17 AIS patients with Cobb angles >90° and flexibility between 20 and 30 %, achieving an average correction of 58.7 %.

In the present study, we report the outcomes of a posterior-only, two-stage surgical approach for AIS patients presenting with curves greater than 100° and flexibility under 30 %. This method uses Ponte osteotomies combined with temporary MCGRs, fixed through an innovative construct we have named the “Sistema Quadro.”

## 2 Materials and methods

This retrospective study analyzed patients with severe, rigid Adolescent Idiopathic Scoliosis (AIS) who underwent surgical treatment at our Orthopedic and Spine Unit between July 2019 and July 2024.

Inclusion criteria were as follows:

● diagnosis of AIS classified according to the Lenke classification system (Lenke et al., 2001).
● main curve with a Cobb angle >100° and ≤30 % correction on preoperative supine bending radiographs
● preoperative magnetic resonance imaging (MRI) negative for myeloradicular malformations
● no prior treatment with halo-gravity traction or serial corrective casting
● surgical treatment performed by the same team using a standardized two-stage posterior approach with magnetically controlled growing rods (MCGR; Magec®, NuVasive Specialized Orthopedics, Inc., San Diego, California) and multilevel Ponte osteotomies

A total of nine patients met the inclusion criteria and were included in the analysis.

Written informed consent was obtained from all patients and their legal representatives regarding the use of clinical data for research purposes. The Ethics Committee ‘CESC VR-RO’ of our University Hospital has confirmed that no ethical approval is required. The study was conducted in accordance with the ethical principles of the Declaration of Helsinki and Good Clinical Practice guidelines.

Collected demographic and clinical data included: sex, age, Body Mass Index (BMI), Hospital length of stay (in days), instrumented spinal levels, pedicle screw density, number of Ponte osteotomies, presence of rib resections, intraoperative and postoperative neurological and mechanical complications.

All procedures were performed by an experienced spinal surgery team using C-arm fluoroscopic guidance and intraoperative neuromonitoring, including electromyography (EMG), motor evoked potentials (MEP), and somatosensory evoked potentials (SSEP). Following implantation, the magnetic rods were gradually lengthened daily using an External Remote Controller (ERC).

An expert spine surgeon evaluated all patients at each stage of treatment and analyzed radiographic imaging.

Radiographic parameters assessed included: main curve (MC) Cobb angle, thoracic kyphosis (T5–T12), bisacromial (clavicular) angle, thoracic height (T1–T12), trunk height (T1–S1), MCGR lengthening, coronal and sagittal balance, calculated as the distance between the C7 plumb line (C7PL) and the central sacral vertical line (CSVL).

Curve angles were measured using the Cobb method (Cobb, 1948), and results were reported in degrees (°) for angles, millimeters (mm) for distances and elongations, and percentages (%) for correction rates.

Spinal flexibility was assessed with preoperative supine side-bending radiographs, expressed as the percentage of correction compared to standing anteroposterior (AP) full-spine radiographs.

Clinical outcomes were evaluated using the Scoliosis Research Society-22 (SRS-22) questionnaire.

One patient, initially treated using a two-stage approach involving a thoracoscopic anterior release at the curve apex (T8–T9 and T9–T10) followed by posterior fusion, was excluded from the study as the treatment protocol did not meet the inclusion criteria.

### 2.1 Statistical analysis

Continuous date were reported as mean, minimum, and maximum values. Comparisons between preoperative and postoperative data were performed using the two-tailed Student's t-test. A p-value of <0.05 was considered statistically significant. Correlations between variables were assessed using the Pearson Correlation Coefficient (PCC).

### 2.2 Surgical technique

The fusion area was determined according to the criteria established by Lenke (Lenke et al., 2001).

**Fig. 1:**
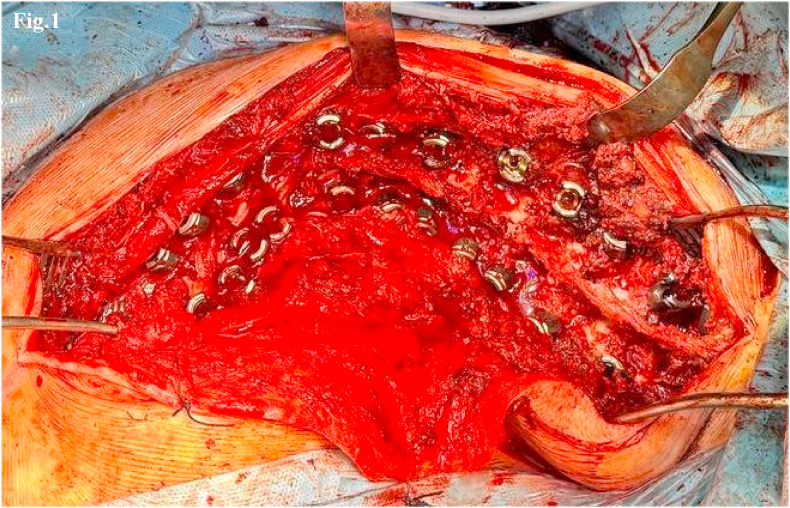
Intraoperative image showing a high-density pedicle screw construct from T3 to L4 for the treatment of a Lenke Type 1 scoliotic curve.

**Fig. 2:**
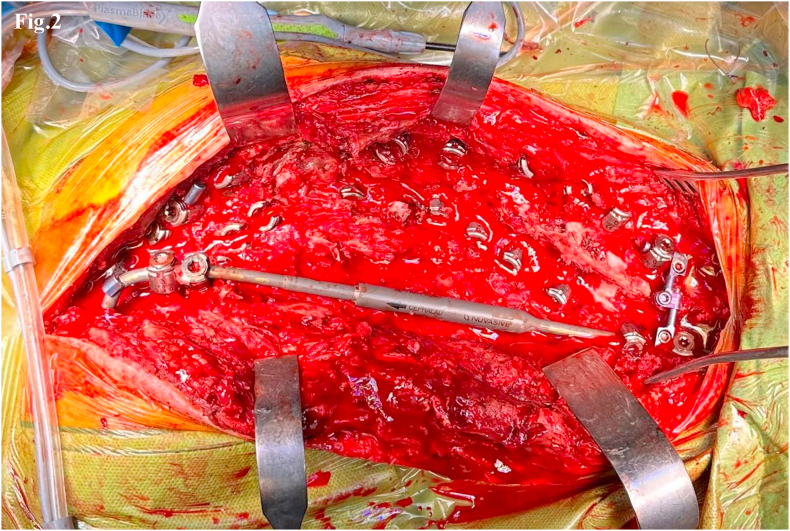
Intraoperative image showing the application of a MCGR on the concave side of a Lenke Type 1 scoliotic curve, anchored at T3–T4 and L3–L4 using the “Sistema Quadro” construct.

During the first posterior surgical stage, a meticulous release of muscle and ligamentous structures, multiple facet joint arthrectomies, and free-hand pedicle screw placement were performed (Fig. 1). Multiple Ponte osteotomies were carried out at the apex of the main curve. A magnetically controlled growing rod (MCGR) was then implanted on the concave side of the curve (Fig. 2). To enhance derotation force transmission to the vertebral bodies, uniplanar pedicle screws were used at the apical vertebrae. Ponte osteotomies were typically performed at two vertebral levels above and below the apex to maximize corrective potential and allow for progressive distraction.

The MCGR was anchored to the spine using a rigid double-fixation construct called the “Sistema Quadro” (Fig. 2). This configuration involved securing the MCGR with at least four pedicle screws at the upper and lower instrumented vertebrae (UIV and LIV). The “Sistema Quadro” consisted of: the MCGR secured to the spine via at least two screws on the concave side, a short rod fixed to two contralateral screws on the convex side, a transverse cross-link connector joining the concave and convex rods, forming a quadrangular frame at both UIV and LIV.

After fixation, mechanical distraction using forceps was performed proximally and distally, representing the first phase of deformity correction. Morcellized autologous bone was grafted at the distal surgical site. A subfascial drain was placed, and the wound was closed in multiple layers with a continuous intradermal suture for the skin.

From postoperative day one, daily MCGR lengthening was carried out using the External Remote Controller (ERC) for approximately two weeks. Each lengthening was continued until the maximum torque threshold was reached or a characteristic “clunking” sound was observed, indicating rod extension. This constituted the second phase of deformity correction. A full-spine radiograph was obtained at the beginning of the lengthening phase, followed by daily ultrasound monitoring. A final radiograph was taken upon completion of the distraction period.

During the second surgical stage, the transverse connectors and the short contralateral rods were removed, with the MCGR temporarily left in place. A pre-contoured cobalt-chrome or titanium rod was inserted on the convex side and seated into the tulips. The MCGR was then explanted and replaced with a second pre-contoured rod on the concave side. The concave rod was more aggressively contoured than the convex rod to produce an indirect derotation effect. Both rods were asymmetrically molded and rotated to achieve a synergistic correction of the sagittal profile, particularly enhancing thoracic kyphosis and lumbar lordosis.

Rotation tubes were applied to the apical uniplanar screws on both the concave and convex sides, as well as at the LIV or neutral vertebra, to apply gentle derotation and counter-torque forces. If needed, selective compression and distraction maneuvers were performed before final rod engagement.

**Fig. 3:**
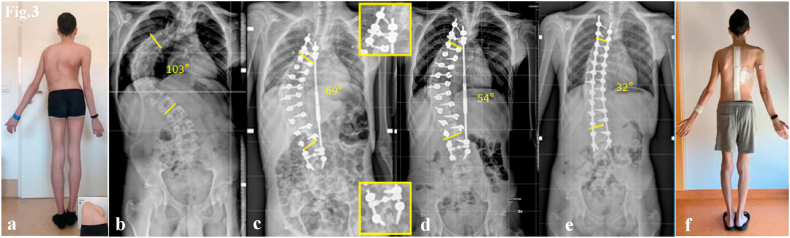
Case 1. 15-year-old male with a Lenke Type 1 curve, treated with T4–L4 posterior spinal fusion. Antero-posterior images show: (a) preoperative clinical assessment with thoracic hump, (b) preoperative radiograph, (c) MCGR implantation prior to distraction and proximal/distal “Sistema Quadro” construct (d) correction after MCGR lengthening, (e) final radiographic correction with definitive rods, (f) final clinical correction.

**Fig. 4:**
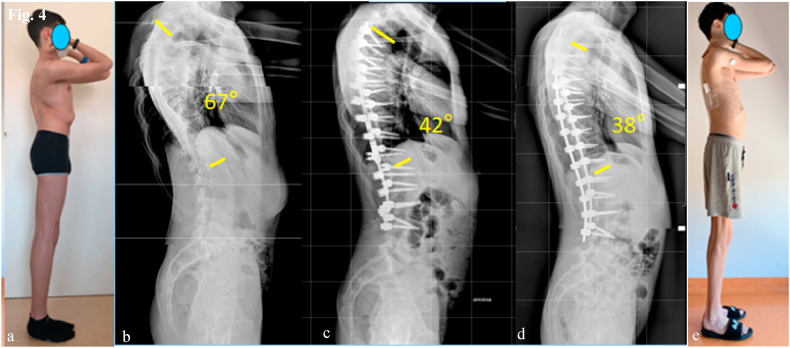
Case 1. Lateral images show: (a) preoperative clinical assessment, (b) preoperative radiograph, (c) correction with MCGR lengthening, (d) final radiographic correction with definitive rods, (e) final clinical correction.

This final procedure represented the third phase of deformity correction (Fig. 3, Fig. 4).

In selected cases, a third reinforcement rod was placed at the apex on the concave side to enhance construct stability.

The procedure concluded with extensive decortication of the posterior spinal elements at all instrumented levels to prepare a solid fusion bed, followed by grafting of morcellized autologous and homologous bone. A subfascial drain was placed, and the surgical wound was closed in multiple layers with a continuous intradermal skin suture.

## 3 Results

The reviewed case series included 9 patients (2 males and 7 females) (Table 1). The mean age at surgery was 15 years (range: 12–22), and the mean BMI was 18.8 kg/m^2^ (range: 16–27.2). The average length of hospital stay was 23.4 days (range: 19–29). Eight patients presented with Lenke type 1 scoliosis, and one with Lenke type 3. All patients had a main curve >100°, with a mean preoperative Cobb angle of 107.6° (range: 100°–116°), and an average flexibility of 21.6 % (range: 15–30) on supine bending tests.

**Table 1:** 

Pt	Sex	Age (years)	BMI	Lenke	Main Curve Cobb angle °	Correction after bending test %	Instrumented level	Screw density	Days of hospitalization
CH	F	13	18.0	1	116	22.4	T3-L4	1.64	22
MAM	F	12	19.4	1	109	18.3	T3-L3	2.00	19
PC	F	14	18.4	1	115	17.4	T3-L4	1.93	26
FSK	M	15	17.9	1	103	20.4	T4-L4	2.00	24
SS	F	16	27.2	3	100	30.0	T2-L4	1.93	22
AM	M	15	16.0	1	110	19.1	T3-L3	2.00	29
BS	F	22	20.0	1	100	15.0	T3-L4	2.00	25
VM	F	15	17.6	1	110	30.0	T4-L4	1.92	24
MC	F	13	15.1	1	105	19.1	T3-L5	1.87	20

Mean		15	18.8		107.6	21.3		1.92	23.4

The upper instrumented vertebrae (UIV) were typically T3–T4 for Lenke 1 and T2–T3 for Lenke 3 curves. The lower instrumented vertebrae (LIV) were L4–L5 for Lenke 1 and L3–L4 for Lenke 3. The mean pedicle screw density was 1.92 (range: 1.64–2.0). A proximal hook was used in one case due to dysplastic pedicles. The mean number of Ponte osteotomies performed was 4.7 (range: 4–6). In three cases, a third reinforcement titanium rod was placed on the concave side of the main curve. The average interval between the two surgical stages was 19.4 days (range: 13–27). The mean clinical follow-up was 31.4 months (range: 7–58), while radiological follow-up averaged 25 months (range: 7–48).

The main curve was corrected from a mean preoperative Cobb angle of 107.6° (range: 102°–116°) to a mean postoperative Cobb angle of 35.4° (range: 30°–46°), representing an average correction of 65.9 % (range: 56.2–74.1). Deformity correction was achieved in three phases (Table 2): phase 1 involved intraoperative distraction through MCGR, contributing 52.5 % of the total correction; phase 2 consisted of daily rod lengthening with the ERC, contributing 24.5 % and phase 3 involved final triplanar correction and fusion, contributing 23 %.

**Table 2:** 

Pt	MC pre-op (°)	MC Post 1° surgery (°)	MC after lengthening (°)	MC final fusion (°)	Correction MC after 1° surgery (%)	Correction MC after MCGR lengthening (%)	Correction MC after 2° surgery (%)
CH	116	62	49	30 (74.1 %)	54° (62.8 %)	13° (15.1 %)	19° (22.1 %)
MAM	109	50	39	31 (71.6 %)	59° (75.6 %)	11° (14.1 %)	8° (10.3 %)
PC	115	75	51	45 (60.9 %)	40° (57.1 %)	24° (34.3 %)	6° (8.6 %)
FSK	103	69	54	32 (68.9 %)	34° (47.9 %)	15° (21.1 %)	22° (31 %)
SS	100	73	60	34 (66 %)	27° (40.8 %)	13° (19.8 %)	26° (39.4 %)
AM	110	72	54	32° (70.9 %)	38° (48.7 %)	18° (23.1 %)	22° (28.2 %)
BS	100	75	53	38° (62 %)	25° (40.3 %)	22° (35.5 %)	15° (24.2 %)
VM	110	72	52	41° (62.7 %)	38° (55.1 %)	20° (29 %)	11° (15.9 %)
MC	105	79	62	46 (56.2 %)	26° (44.1 %)	17° (28.8 %)	16° (27.1 %)

Mean	107,6	69,7	52,7	35.4 (65.9 %)	37.9 (52.5 %)	17 (24.5 %)	16.1 (23 %)

Sagittal profile: thoracic kyphosis decreased in five patients from a mean of 48.8° (range: 20°–81°) to 36.2° (range: 16°–52°), with an average reduction of 25.8 % (range: 24.4–35.0 %).

Spinal height gains (Table 3): trunk height (T1–S1) increased from 35.8 cm (range: 27.2–42.4 cm) to 45.3 cm (range: 38.1–51.2 cm), with a mean gain of 9.5 cm; thoracic height (T1–T12) increased from 22.1 cm (range: 18.1–26.1 cm) to 27.5 cm (range: 24.9–31.9 cm), for a mean gain of 5.4 cm.

**Table 3:** 

Pt	Sex	Age (years)	BMI	Thoracic height (Tx) pre-op (mm)	Trunk height (Tk) pre-op (mm)	Thoracic (Tx) height after 1° surgery (mm)	Trunk (Tk) height after 1° surgery (mm)	N pontes Osteotomy	MCGR lengthening (mm)	Thoracic height (Tx) after lengthening (mm)	Trunk height (Tk) after lengthening (mm)	Thoracic height (Tx) after 2° surgery (mm)	Trunk height (Tk) after 2° surgery (mm)
CH	F	13	18	204	336.7	220.1	386	4	17	240.6	417.7	260	451
MAM	F	12	19.4	203.6	335.4	222.7	370.2	4	12	241.6	398.9	249.6	423.9
PC	F	14	18.4	203.1	321.1	216.3	353	6	22	245.3	398.7	253.3	427.9
FSK	M	15	17.9	239	387.1	279.6	464.4	4	26.1	288.7	480.8	296.7	482.4
SS	F	16	27.2	222.9	387.8	258.6	441.3	4	10.6	285.1	480	286	481.1
AM	M	15	16	261.4	423.8	292.5	479	5	19	307.6	497.45	318.8	512.4
BS	F	22	20	237.4	391.1	250	449	5	18	265.4	450.7	277.4	456
VM	F	15	17.6	236	368	268	447.1	4	21	274.6	453.4	279	462.2
MC	F	13	15.1	180.5	272	215.1	343.2	6	27.3	241	363.2	253.1	380.8

Mean		15	18.8	220.9	358.1	247	414.8	4.7	19.2	265.54	437.9	274.9	453.1

No significant changes were observed between immediate postoperative and final follow-up measurements.

The bisacromial (clavicular) angle improved from a mean of 4.4° (range: 1°–7°) preoperatively to 0.8° (range: 0°–2°) at final follow-up. Coronal and sagittal balance: on the coronal plane, the C7 plumb line to CSVL distance decreased from 25.7 mm (range: 4.1–72 mm) to 14.4 mm (range: 0–26.9 mm); on the sagittal plane, the C7PL-CSVL distance changed slightly from 12 mm (range: 0–46.35 mm) to 14.3 mm (range: 0–37.02 mm).

Mean absolute value (MAV) of coronal balance was 15.1 mm (range: 0–28.6 mm); sagittal balance MAV was 21.7 mm (range: 0–56.1 mm). Although these measures worsened slightly at final follow-up, the differences were not clinically significant. Patient-reported outcomes: SRS-22 scores improved in all patients, increasing from a preoperative mean of 3.3 (range: 2.3–4.0) to 4.4 (range: 3.8–4.7) at final follow-up (Table 4).

**Table 4:** 

Pt	Clinical FU (months)	Radiological FU (months)	MAV Coronal Balance Preop (mm)	MAV Coronal Balance Postop (mm)	MAV Coronal Balance FU (mm)	MAV Sagittal Balance Preop (mm)	MAV Sagittal Balance Postop (mm)	MAV Sagittal Balance FU (mm)	Clavicle Angle Preop (°)	Clavicle Angle Postop (°)	Clavicle Angle FU (°)	Thoracic Kyphosis Preop (°) (T4-T12)	Thoracic Kyphosis Postop (°) (T4-T12)	Thoracic Kyphosis FU (°) (T4-T12)	SRS22 Preop	SRS22 Postop
CH	58	48	21.0	4.0	17.1	11.42	0.0	56.0	6.7	1.0	1.4	47	41	35	3.3	3.8
MAM	56	47	25.2	7.2	2.6	35.0	0.0	0.0	5.0	2.0	1.4	20	16	18	2.3	4.6
PC	47	39	72.0	0.0	26.0	46.3	37.0	23.0	5.0	2.0	2.7	81	43	36	3.5	3.9
FSK	34	25	13.6	26.9	19.6	4.3	23.4	3.5	4.2	0.0	3.3	67	38	49	4.0	4.5
SS	26	13	20.0	16.7	18.1	0.0	7.14	27.7	1.0	1.0	7.0	55	38	39	3.0	4.6
AM	25	16	42.0	4.3	0.0	15.4	17.3	0.0	7.0	0.0	1.0	42	42	26	3.3	4.5
BS	16	16	5.3	26.0	28.6	1.2	26.2	12.2	3.0	0.0	1.0	53	52	42	4.0	4.7
VM	14	14	22.7	18.0	4.2	25.5	0.0	56.1	3.0	0.0	1.4	49	40	37	2.6	4.6
MC	7.0	7.0	4.1	26.6	19.9	0.0	17.3	16.7	5.0	1.0	2.0	25	16	24	3.3	4.5

Mean	31.4	25	25.7	14.4	15.1	12.0	14.3	21.7	4.4	0.8	2.4	48.8	36.2	34	3.3	4.4

Pre-operative, post-operative and final follow-up clinical and radiographic measurements.

A statistically significant inverse correlation was found between BMI and total rod lengthening (PCC = −0.7304; p = 0.026). A positive correlation was observed between the number of osteotomies and rod lengthening (PCC = 0.52; p = 0.154), although it did not reach statistical significance (Table 3).

No intraoperative or postoperative mechanical complications (e.g., screw pull-out, pedicle fractures) or neurological complications (transient or permanent) were observed.

**Fig. 5:**
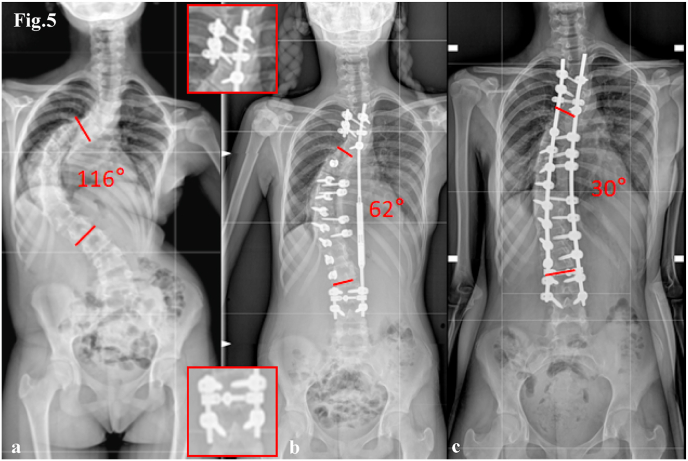
Case 2. 13-year-old female with a Lenke Type 1 curve, treated with T3–L4 posterior spinal fusion. Antero-posterior radiographs show: (a) preoperative curve, (b) correction with MCGR lengthening and proximal/distal “Sistema Quadro” construct, (c) final correction with definitive titanium/cobalt–chrome rods.

**Fig. 6:**
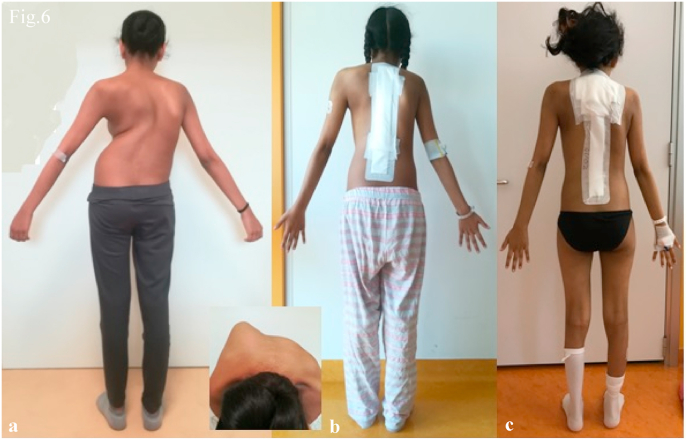
Case 2. Antero-posterior clinical images show: (a) preoperative curve with thoracic hump, (b) correction with MCGR, (c) final correction with definitive titanium/cobalt–chrome rods.

**Fig. 7:**
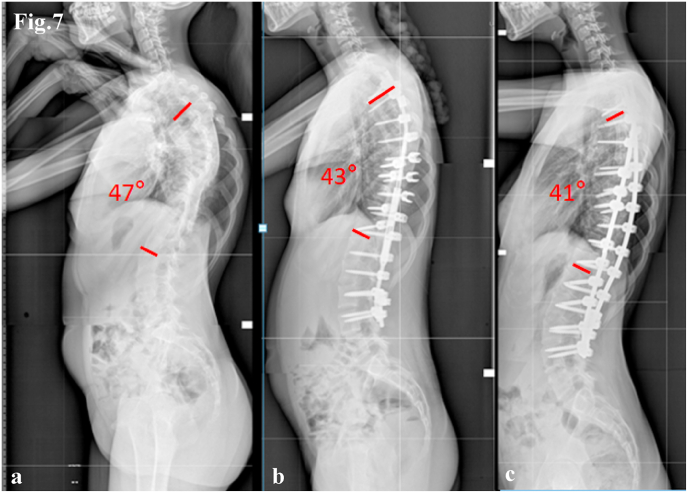
Case 2. Lateral radiographs show: (a) preoperative curve, (b) correction with MCGR, (c) final correction with definitive titanium/cobalt–chrome rods.

**Fig. 8:**
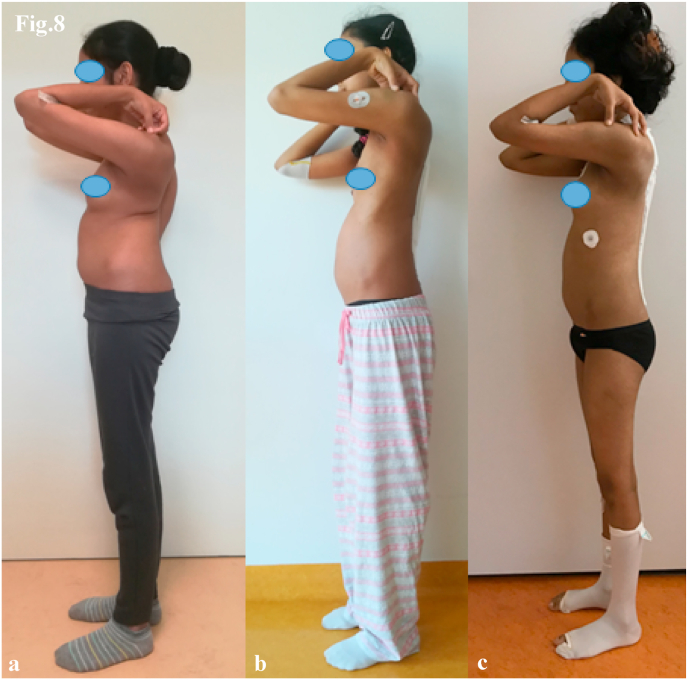
Case 2. Lateral clinical images show: (a) preoperative curve, (b) correction with MCGR, (c) final correction with definitive titanium/cobalt–chrome rods.

Representative cases are illustrated in Fig. 3, Fig. 4, Fig. 5, Fig. 6, Fig. 7, Fig. 8.

## 4 Discussion

The surgical management of severe and rigid adolescent idiopathic scoliosis (AIS) remains a significant challenge for spine surgeons. The primary goal is to achieve optimal clinical and radiographic outcomes while minimizing surgical risk and preserving patient safety. Recent evidence supports a two-stage posterior approach using a magnetically controlled growing rod (MCGR), in combination with multiple Ponte osteotomies at the apex of the deformity, as a safe and effective strategy for this complex condition.

Di Silvestre et al. (Di Silvestre et al., 2020), in a multicenter study of 17 cases with major curves exceeding 90° and flexibility between 20 and 30 %, reported a mean final correction of 58.7 % without permanent neurological complications. Similarly, Koller et al. (2021) treated seven patients at two specialized centers with curves >100° and flexibility ≤30 %, achieving a mean correction of 67 %, again with no permanent neurological sequelae. In our single-center series of nine patients with comparable curve magnitude and rigidity, we achieved a correction exceeding 65 %, with no mechanical failures and no transient or permanent neurological complications. These consistent outcomes across studies reinforce the efficacy and safety of the two-stage posterior MCGR-based technique for managing severe and rigid AIS.

A major benefit of this technique is the restoration of thoracic and trunk height, reducing the mismatch between trunk height and leg length caused by severe spinal deformity In our series, we observed substantial gains in spinal height—mean thoracic length (T1–T12) increased by 4.67 cm and trunk height (T1–S1) by 9.5 cm—alongside a mean Cobb angle reduction from 107.6° to 35.4°. Such structural and cosmetic results would not be achievable using single-stage posterior-only techniques based on vertebral column resection (VCR), which carry higher risk and morbidity.

Unlike Di Silvestre and Koller, our study introduced an innovative MCGR anchoring construct, the “Sistema Quadro”, which provides both mechanical and functional advantages. The MCGR is anchored to the upper and lower instrumented vertebrae (UIV and LIV) via two rigid fixation blocks. Each block distributes corrective forces across at least four screws, reducing stress on individual pedicles and minimizing the risk of screw pull-out or fracture. This stabilization is particularly advantageous in the presence of thoracic hyperkyphosis or poor bone quality. Furthermore, the bilateral “square” configuration ensures balanced force distribution, improving coronal alignment and shoulder balance, while optimizing the spine's preparation for final correction (Fig. 3, Fig. 5).

Neurological complications during scoliosis correction are often related to mechanical stress or ischemia of the spinal cord caused by aggressive correction maneuvers (Biscevic et al., 2020; Tong et al., 2019). The risk increases significantly in patients with curves >90° (Feng et al., 2012; Da et al., 2022). In a multicenter analysis, Buckwalter et al. (2013) identified a higher incidence of intraoperative neuromonitoring alerts in cases with large, rigid upper thoracic curves. These findings highlight the importance of controlled, gradual correction.

Neurological complications, both transient and permanent, have been reported in staged corrections (0–14.2 %) (Di Silvestre et al., 2020; Koller et al., 2021; Hwang et al., 2020; Yamin et al., 2008; Koptan and ElMiligui, 2012; Sponseller et al., 2008; Watanabe et al., 2010) and in single-stage procedures involving VCR (0–27 %) (Suk et al., 2005; Lenke et al., 2013; Zhou et al., 2011). Even the innovative Hi-PoAD technique (High-Density Pedicle Screws, Ponte Osteotomies, Asymmetric Rod Contouring, Direct Vertebral Rotation), proposed by Faldini et al. (2023) for severe AIS, was associated with a 15.8 % rate of transient neurological complications, necessitating interruption of the surgical procedure, with the surgical team resuming the operation after five days.

Our experience with an initial anterior thoracoscopic release and posterior MCGR-assisted correction (excluded from this review) confirmed literature findings on the higher complication rates of single-stage approaches (Spina et al., 2020). Following the failure of the anterior thoracoscopic release and partial correction with the magnetic rod, the second posterior surgical stage transformed into a single-stage procedure. During this surgical procedure, significant reductions in neurophysiological signals (Somatosensory Evoked Potentials-SEP and Motor Evoked Potentials-MEP) were observed, with recovery achieved only after reducing screw density and relying on the partial correction already obtained.

In contrast, our standardized three-phase posterior-only strategy demonstrated both safety and effectiveness (Table 2). The first phase involved gentle intraoperative distraction using the MCGR, achieving 52.5 % of the total correction. This was followed by daily rod lengthening with the ERC during the second phase, accounting for 24.5 % of correction. The final phase consisted of triplanar correction and fusion, contributing the remaining 23 %. This gradual, staged approach allows the spinal cord to adapt progressively to alignment changes, minimizing the risk of neurological injury.

Historically, gradual deformity correction has also been achieved with halo-gravity traction, either alone (Hwang et al., 2020) or following anterior (Yamin et al., 2008; Koptan and ElMiligui, 2012; Sponseller et al., 2008; Watanabe et al., 2010) or posterior release (Mehlman et al., 2004). While halo traction improves curve flexibility and respiratory function (Sun et al., 2023), its limitations include prolonged hospitalization (up to 70.1 days) (Bogunovic et al., 2013) and high complication rates (up to 94.7 %) (Popescu et al., 2022), including pin-site infections, neurological symptoms, and pain. These factors significantly limit its feasibility and patient acceptance.

The MCGR-based technique was well tolerated by our patients. Postoperative rehabilitation began with bedside physiotherapy on day one, sitting on day two, and ambulation on day three. Six of nine patients were discharged home after completing the MCGR lengthening phase while awaiting definitive fusion. These findings suggest that, in selected cases, outpatient rod lengthening may be feasible, offering potential reductions in hospitalization time and healthcare costs.

Strengths of our study include a homogeneous patient population, standardized surgical technique, and dedicated surgical team in a single center. Limitations include the small sample size and variability in follow-up duration (7–58 months), which reflect the rarity of such extreme deformities in developed healthcare settings.

## 5 Conclusions

Our results demonstrate that a two-stage posterior-only approach utilizing MCGR and multiple Ponte osteotomies is a safe, effective, and well-tolerated treatment for AIS patients with curves exceeding 100° and flexibility of 30 % or less. The use of the “*Sistema Quadro*” along with the *three-phase correction* approach significantly contributed to these positive results. Given its benefits, this technique should be more widely adopted by spine surgeons managing complex spinal deformities. Further studies are necessary to assess its long-term cost-effectiveness and broader applicability.

## Consent to participate

Informed consent was obtained from all individual participants included in the study.

## Consent to publish

The authors affirm that human research participants provided informed consent for publication of all the images.

## Disclosures

None.

## Author contributions

All authors contributed to the study conception and design. Material preparation, data collection and analysis were performed by E.S.D. and R.G. The manuscript was written by M.S and F.G. The patients were operated by M.B. and M.S. All authors have read and agreed to the published version of the manuscript.

## Ethics approval

This study was performed in line with the principles of the Declaration of Helsinki. This is an observational study. The Ethics Committee “CESC VR-RO” of Azienda Ospedaliera Universitaria Integrata di Verona (Italy) has confirmed that no ethical approval is required.

## Funding

The authors declare that no funds, grants, or other support were received during the preparation of this manuscript.

## Conflict of interest

The authors declare no competing interests.
